# Workplace violence toward emergency medicine physicians in the hospitals of Taif city, Saudi Arabia: a cross-sectional survey

**DOI:** 10.1186/s12873-022-00620-w

**Published:** 2022-04-07

**Authors:** Yasser H. Alnofaiey, Fahad M. Alnfeeiye, Osama M. Alotaibi, Anas A. Aloufi, Saud F. Althobaiti, Abdulmajeed G. Aljuaid

**Affiliations:** 1 Department of Emergency Medicine, Taif University, Taif, Saudi Arabia; 2 College of Medicine, Taif University, Taif, Saudi Arabia

**Keywords:** Emergency department, Hospital, Physicians, Workplace violence

## Abstract

**Background:**

Workplace violence against health care workers is an emerging concern in various global health settings and the documentation of physical and verbal attacks against physicians in tertiary hospitals in Saudi Arabia is uncommon. This study aimed to determine the incidence of workplace violence against physicians in the emergency department of selected tertiary hospitals in Taif City, Saudi Arabia from June to July 2021. Associations between the incidence of violence and interventions and type of physicians and years of experience were also investigated.

**Methodology:**

Using a cross-sectional design, a total of 96 physicians were recruited to answer the World Health Organization Questionnaire on Violence against Health Care Workers last June to July 2021.

**Results:**

It was found out that 75 physicians (78.1%) experienced verbal violence while 14 physicians (14.6%) experienced physical violence. Most of the workplace violence happened within hospital premises (84.4%). Despite having an experience of verbal and physical violence, only 44.8% of the respondents reported the incidents. The most common instigators were patients (55.21%), their relatives (78.00%), external colleagues (9.37%) and staff members (6.25%). Most respondents took no action, or reported the incidence to the police or senior staff member. The incidence of workplace violence and type of physician showed significant association. The number of years of practice in emergency medicine also exhibited statistically significant association with the incidence of verbal attack, frequency of violence, and location of incident.

**Conclusion:**

There is high incidence of workplace violence among physicians in tertiary hospitals, and younger physicians with less experience in emergency medicine were the most susceptible to both verbal and physical violence. There is a need to strengthen policies to protect physicians against workplace violence.

## Introduction

Workplace violence is a common experience among health care workers, and is considered as an emerging issue to be addressed in various global healthcare contexts [1]. In several health settings, workplace violence is considered a major occupational and health hazard which requires early prevention or mitigation [2, 3]. The World Health Organization defines workplace violence as the “*intentional use of physical force or power, threatened or actual, against oneself, another person, or against a group or community that either results in or has a high likelihood of resulting in injury, death, psychological harm, mal-development, or deprivation*” [4]. Currently, workplace violence is considered as a public health problem [5, 6]. In most cases, workplace violence involved physical attacks, verbal abuse and initiation of mental stress. Reports on workplace violence has included several health care workers such as nurses [7–11], emergency medical technicians [12], and physicians [5, 13, 14]. In Saudi Arabia, there is a lack of policy which promotes the protection health care workers from workplace violence which is instigated by patients.

According to the WHO, workplace violence can involve physical and psychological violence, both of which leads to decline in workplace productivity and health care quality [1]. In addition, workplace violence has been reported to be related to job burnout, job dissatisfaction, and turnover intention [2]. It was reported that in the health care settings, the most common types of violence reported include verbal abuse, threatening behaviors, physical assaults, and sexual harassment [8]. In either case, violence against health care workers leads to a negative effect on both physical and psychological well-being in various health settings [5].

The most common setting of physical and psychological violence is the hospital [9, 15–17]. In various studies, the perpetrators of workplace violence included patients and visitors [15, 17, 18], and other workers [17, 19]. Some of the most common instigating causes of workplace violence include failure to meet patient expectations [5, 7] and deficient staff number [4] which lead to patient aggression. While it is expected that patient dissatisfaction should encourage feedback towards the improvement of health care services in the emergency department, there is also a possibility that the stressful environment in the health setting may initiate incidence of workplace violence.

In Saudi Arabia, initial efforts in assessing the incidence of workplace violence have been documented among health care workers. Violence towards health care workers have been documented among nurses in the emergency department in Riyadh [7], with most cases associated with verbal abuse while physical abuse was relatively uncommon. Violence against health care workers situated in primary care centers in Al Khobar, Eastern Province has also been documented [20], with most cases attributed to verbal violence and intimidation. However, the incidence of workplace violence among physicians working in the emergency department in Taif City, Saudi Arabia is still undocumented. In another study, it was reported that the most common form of workplace violence in the emergency department of certain hospitals in Saudi Arabia were related to verbal attacks [5] and the most susceptible health care professionals were physicians. In the study of [17], it was revealed that nurses in Saudi Arabia who experienced workplace violence did not report the incidence due to fear for negative consequences and feelings of uselessness.

With the pervasiveness of workplace violence, the manner how the issue on physical and verbal violence was resolved between health care professionals and the perpetrators was often remarked to be unsatisfactory [17]. The dismal effort towards protecting health care professionals against violence in the workplace may compromise the well-being of health care professionals, leading to poor performance and consequent work dissatisfaction. In Saudi Arabia, issues on work and may require further investigation to promote the well-being of the health care worker and the patient.

Hence, this study sought to investigate the incidence of physical and verbal violence in selected hospitals in Taif City, Saudi Arabia, and the interventions done by the physicians to mitigate workplace violence. In addition, associations between the incidence of violence and interventions done with the type of physicians, and type of physicians and years of experience in the emergency medicine department were also investigated. The results of the study can serve as a guide to the development of relevant hospital policies which can address workplace violence in the emergency department of hospitals in Taif City, Saudi Arabia. The study’s aim to determine the types of workplace violence and types of perpetrators in the workplace can provide context-specific policies that can address not only the concerns of residents and physicians, but other health care workers as well. Furthermore, the information gathered on the current intervention utilized by physicians in the emergency department of hospitals to address the incidence of verbal and physical violence can also be utilized to draft additional guidelines on national policies which protects the rights of health care professionals.

### Subjects and methods

This study utilized a cross-sectional research design using an adapted research instrument, duly adapted for online data gathering. Physician respondents who work in the emergency department were recruited from Taif City, Saudi Arabia. The inclusion criteria of respondents included: a) currently employed physician in a hospital in Taif city, b) male or female, c) works in morning, afternoon or night shifts, and d) willing to participate in the study. For the exclusion criteria, any respondent who refused to sign the informed consent prior to answering the research instrument, or who are unable to answer the test due to any health condition, or those who have other conditions which might influence their performance on the test were excluded from the study. All respondents who did not have any internet access and expressed withdrawal from answering the questionnaire were also excluded from the analysis of data.

This study gathered data using the WHO Questionnaire on Violence against Health Care Workers. The research instrument is divided into three parts. The first part obtained the sociodemographic data of the physicians while the second part determined the respondents’ experiences with violence in the workplace. The third part identified the factors which may lead to violence and strategies for the prevention of violence in workplace, as perceived by the respondents.

The study was approved by the Institutional Ethics Committee of the Directorate of Health Affairs in Taif, Saudi Ministry of Health (MOH), Research Protocol # HAP-02-T067. Anonymity was ensured by assigning a code for each respondent. All files were secured in a single document to ensure confidentiality of information. Using systematic sampling, a total of 120 residents and physicians were recruited and 96 residents and physicians who received an online link to an electronic version of the research instrument completely answered all questions, which is equivalent to 80% response rate. All respondents were physicians who had worked in the emergency department. The invitation to answer the questionnaire was sent to the respondents’ email, after seeking permission from the hospitals to obtain their email addresses. Participation to the study was voluntary and complied with the set guidelines by the ethical approval committee and indicated voluntary participation by answering the “Yes” option in the electronic form.

### Data analysis

The data obtained were entered into Microsoft Excel. Quantitative analysis was conducted by summarizing data into frequency and percentage. Chi square (χ^2^) analysis was performed to determine association of variables using SPSS 27.0 (NY, Armonk). Fisher Exact test was utilized in cases where chi square statistics was not appropriate. Bonferroni adjustment was used as post hoc test for associations which were statistically significant. Statistical significance was evaluated at α = 0.05. All *p* values less than 0.05 were considered statistically significant.

## Results

### Demographic characteristics

Table 1 shows the demographic characteristics of the respondents. Based on the results, majority of the respondents in this study are male physicians, comprising 74.0% of the total population. The age range of the respondents is relatively young, with majority belonging to less than or equal to 30 years old (53.1%), followed by 31 to 40 years old (33.3%). Most respondents speak Arabic (92.7%), while the rest speak English (7.3%). Most of the respondents are also junior residents, comprising 41.7% of the total population. This is followed by senior residents (31.3%), and staff physicians (17.7%).

**Table 1:** Descriptive statistics of demographic variables (*n* = 96)

Variable	Category	N	%
**Gender**	Male	71	74.0%
	Female	25	26.0%
**Age**	Less than or equal to 30 years old	51	53.1%
	31 to 40 years old	32	33.3%
	41 to 50 years old	9	9.4%
	Greater than or equal to 51 years old	4	4.2%
**Spoken Language**	Arabic	89	92.7%
	English	7	7.3%
**Type of Physician**	Junior Resident	40	41.7%
	Senior Resident	30	31.3%
	Staff Physician	17	17.7%
	Assistant Consultant	3	3.1%
	Consultant	6	6.3%
**Years of Practice in Emergency Medicine**	5 years or less	64	66.7%
	6 to 20 years	30	31.3%
	21 to 30 years	2	2.1%
**Emergency medicine board certification**	No	68	70.8%
	Yes	28	29.2%
**Type of Hospital**	Governmental (MOH)	85	88.5%
	Governmental (Non-MOH)	10	10.4%
	Private	1	1.0%
**Residency Program**	No	26	27.1%
	Yes	70	72.9%

Based on the experience in emergency medicine, a large number of the respondents had 5 years or less experience (66.7%), while some had 6 to 20 years of experience (31.3%). Most respondents had no emergency medicine board certification (70.8%) and a large majority work in governmental (MOH) hospital. Lastly, most respondents have completed residency programs (72.9%).

Table 2 shows the incidence of workplace violence and the measures done by the physicians to address both physical and verbal attacks in the emergency department setting. Based on the results, it was found out that a large majority of the respondents did not experience physical attacks (84.4%), although majority experienced verbal attacks (78.1%). The frequency of physical or verbal violence varied, although most respondents experienced the incidence of violence in the workplace about 2 to 3 times (29.2%) or more than 5 times (30.2%). Most cases of physical or verbal violence happened inside the hospital (84.4%). However, the respondents indicated that they adhere to the procedure for reporting violence in the workplace (82.3%). While majority of the respondents (44.8%) report the incidence of violence, other respondents felt that reporting the incidence of violence is useless (29.2%).

**Table 2:** Incidence of workplace violence and interventions done by the respondents

Variable	Category	N	%
Physical attack in the workplace (Last 12 months)	No	81	84.4%
	Yes	14	14.6%
	Yes, with weapon	1	1.0%
		N	%
Verbal attack in the workplace (Last 12 months)	No	21	21.9%
	Yes	75	78.1%
Frequency of physical or verbal violence	Once	12	12.5%
	2 to 3 times	28	29.2%
	4 to 5 times	15	15.6%
	More than 5 times	29	30.2%
	Never	12	12.5%
Location of incident	Did not take place	13	13.5%
	Inside the hospital	81	84.4%
	Both inside and outside the hospital	2	2.1%
Procedure for reporting incidence in the workplace	No	8	8.3%
	Yes	79	82.3%
	I don’t know	9	9.4%
Reporting of violence	No, because I am afraid of negative consequences	6	6.3%
	No, because I feel reporting the violence incidence is useless	28	29.2%
	No, because I don’t know whom to report	2	2.1%
	Yes	43	44.8%
	Other reasons	17	17.7%

Table 3 shows the association of the incidence of physical and verbal violence, and interventions done by the physicians to the type of physicians who were included in this study. Based on the results, there is a significant association between the type of physician, and the reporting of the incidence of violence in the workplace. It can be observed that almost all physicians do report the incidence of physical and verbal attack in the workplace.

**Table 3:** Association of incidence of violence and interventions done with the type of physicians

Variable	Category	Type of Physician					χ2	***p***
		Junior Resident	Senior Resident	Staff Physician	Assistant Consultant	Consultant		
Physical attack in the workplace (last 12 months)	No	31	29	13	3	5	10.657	0.222
	Yes	9	0	4	0	1		
	Yes, with weapon	0	1	0	0	0		
Verbal attack in the workplace (last 12 months)	No	7	7	3	2	2	4.646	0.326
	Yes	33	23	14	1	4		
Frequency of physical or verbal violence	Once	7	2	2	0	1	18.640	0.288
	2 to 3 times	12	12	3	0	1		
	4 to 5 times	8	3	4	0	0		
	More than 5 times	9	9	7	1	3		
	Never	4	4	1	2	1		
Location of incident	Outside the hospital	4	5	1	2	1	11.543	0.173
	Inside the hospital	34	25	16	1	5		
	Both inside and outside the hospital	2	0	0	0	0		
Knowledge of procedure for reporting incidence in workplace	No	2a	1a	4a	1a	0a	22.700	**0.004**
	Yes	30a,b	29b	13a,b	1a	6a,b		
	I don’t know	8a,b	0b	0a,b	1a	0a,b		
Reporting of violence	No, because I am afraid of negative consequences	1	1	3	0	1	24.490	0.079
	No, because I feel reporting the violence incidence is useless	13	7	5	1	2		
	No, because I don’t know whom to report	0	1	0	1	0		
	Other reasons	9	5	2	0	1		
	Yes	17	16	7	1	2		

Post-hoc test used was Bonferroni adjustment. Data assigned with different letters indicate significant difference of column proportions at α = 0.0

Table 4, on the other hand, shows the association between the incidence of physical and verbal violence, and interventions done by the physicians with the number of years of practice in emergency medicine among physicians. It can be observed that the number of years of practice in emergency medicine is statistically significantly associated with the incidence of verbal attack for the past 12 months, frequency of physical or verbal violence, and location of the incident of violence.

**Table 4:** Association of incidence of violence and interventions done with the year of practice in emergency medicine

Variable	Category	Years of practice in Emergency Medicine			χ2	***p***
		5 years or less	6 to 20 years	21 to 30 years		
Physical attack in the workplace (last 12 months)	No	52	27	2	1.766^a^	0.779
	Yes	11	3	0		
Verbal attack in the workplace (last 12 months)	No	15a	4a	2b	8.515	**0.014**
	Yes	49a	26a	0b		
Frequency of physical or verbal violence	Once	9a	3a	0a	19.912	**0.011**
	2 to 3 times	18a	10a	0a		
	4 to 5 times	12a	3a	0a		
	More than 5 times	16a	13a	0a		
	Never	9a	1a	2b		
Location of incident	Outside the hospital	10a	1a	2b	16.853	**0.002**
	Inside the hospital	52a	29a	0b		
	Both inside and outside the hospital	2a	0a	0a		
Knowledge of procedure for reporting incidence in workplace	No	6	2	0	2.800	0.592
	Yes	50	27	2		
	I don’t know	8	1	0		
Reporting of violence	No, because I am afraid of negative consequences	2	4	0	10.841	0.211
	No, because I feel reporting the violence incidence is useless	17	11	0		
	No, because I don’t know whom to report	2	0	0		
	Other reasons	15	2	0		
	Yes	28	13	2		

Post-hoc test used was Bonferroni adjustment. Data assigned with different letters indicate significant difference of column proportions at α = 0.05

The respondents revealed that the relatives (*N* = 78, % = 81.25), patients (*N* = 53, % = 55.21), external colleagues (*N* = 9, % = 9.37), staff members (*N* = 6, % = 6.25), as perpetrators of physical or verbal violence in the workplace. The results indicate that some residents and physicians have experienced workplace violence from multiple perpetrators. When asked on their response to the incidence of violence, 20.83% responded that they took no action, 20.83% reported the incidence to the police and 17.71% reported it to their senior staff member. It is noteworthy to report in this study that 20.83% also took no action, and the rest had no experience of violence.

## Discussion

The emergency department is one of the most stressful health environments in the hospital, and health workers in the emergency department face threats to safety and security due to workplace violence. Physicians are among the health care workers who often experience workplace violence [9, 14, 21], and only a handful of literature has studied the dynamics of workplace violence among physicians in the emergency department. While it is acknowledged that collaborative and efforts have been initiated to mitigate workplace violence in hospital settings [5, 7–13], much remains to be investigated in the emergency department of hospitals in Saudi Arabia.

The multidisciplinary nature of task required in emergency cases justifies physicians as the most important component of the health care team in the emergency department in hospitals [22]. Physicians in the emergency department deal with a diverse type of cases which require prompt or immediate intervention, and may require flexibility to prioritize patient needs while ensuring safety. While it is common that patient dissatisfaction allows protection against negligence and malpractice in the emergency department, and requires health professionals to adhere to the highest standard quality of care, patient dissatisfaction might also result to unnecessary incidences such as physical and verbal attacks against health care professionals in the workplace. It is claimed in this study that workplace violence is indeed common in the hospital setting, similar to previous reports [15–17]. This study showed there was a high prevalence of workplace violence against physicians in the Emergency Department of hospitals in Taif City, Saudi Arabia, with verbal abuse, and physical violence the most common types of incidents. The main perpetrators of workplace violence were patients or their relatives. However, this study was not able to further identify the profile of patients who commonly instigate workplace violence.

Physicians are among the most important components of the emergency department, and the same can be concluded in the large hospitals of Saudi Arabia. An earlier report has revealed that physicians in Saudi Arabia were the most exposed to verbal and physical violence in the workplace [5]. Similarly, other studies have also reported that verbal attacks were commonly experienced by physicians in various hospital settings [9, 14, 21]. Since physicians are the most important component of the health care team in the emergency department, but exhibit susceptibility to work-related stress and possible job dissatisfaction related to workplace violence, there is a possibility that physicians might exhibit poor performance amidst the threat to their safety and morale in the emergency department [2]. The aforementioned rationale enabled the study to document the incidence of workplace violence against physicians in hospitals of Taif City, Saudi Arabia.

The results of this study agree with other studies which have been conducted earlier in Saudi Arabia. Most experiences related to workplace violence in Saudi Arabia [5, 7] were related to verbal attacks (Table 2). While gender was previously reported to be associated to the type of violence experienced by healthcare workers in the workplace [7, 18], the results of this study did not find any statistically significant association with the type of workplace violence and interventions done to mitigate the either verbal or physical abuse with the gender of the physicians. This could be related to the relatively lower number of female respondents who were recruited in this study. However, the data also suggests that the incidence of physical and verbal abuse might be present in both male and female physicians, but in varying degrees, types and contexts.

There are other interesting findings in this study which need to be explained, in the context of the incidence of physical and verbal forms of violence in the emergency departments. It was noted that there were statistically significant associations between the number of years of practice in emergency medicine with the incidence of verbal attack in the workplace, frequency of workplace violence, and location of the incidence for workplace violence. The data suggests that in the current environment in the emergency department in Taif City, Saudi Arabia, physicians who have 5 years or less experience in emergency medicine were the most susceptible to workplace violence in hospital settings. Younger physicians may be viewed as less competent and are easier targets for workplace violence possibly due to unfamiliarity to the environment, lack of mitigating skills and fewer job-related experiences [1, 5]. It can also be noted in the data that physicians with fewer experience in emergency medicine are more likely to commit diagnostic errors or choice of intervention in a stressful environment, and may have compromised patient safety and subsequence service in the emergency department, as supported by an earlier report [1].

In contrast to previous studies about the healthcare workers being indifferent and reporting nothing to mitigate workplace violence in Saudi Arabia [17, 20], the emergency department physicians in this study exhibited greater assertiveness in addressing both physical and verbal attacks in the workplace by reporting them to senior staff members. While more than 20% of abusive events are unreported, the most common reasons were related to a low threat that is perceived from patient-instigated workplace violence. This implies that the current practice in the hospitals in in the city of Taif, Saudi Arabia involves the encouragement of reporting incidents of workplace violence in the hospital. However, there is a need to elaborate the procedures and mechanisms on how workplace violence is resolved, in favor of the health care worker who experienced violence. In addition, there is dearth of literature on the current sanctions that are given to patients who instigate workplace violence in the institution.

Similar to other studies, the most common instigators of workplace violence against physicians in the emergency department were patients, relatives of patients, and coworkers [15, 17–19]. While it is acknowledged that the emergency department is innately stressful for patients and their family members [22], healthcare workers also share the same predicaments in attending to the needs of the patients while facing the possibility of being susceptible to workplace violence. With the results, it is important to reiterate that workplace violence needs to be addressed, and strategies to capacitate hospitals in protecting the healthcare workers from physical and verbal attacks need to be strengthened [23].

## Conclusion

Workplace violence against physicians is a serious issue to be addressed in the emergency department in selected hospitals in Saudi Arabia. The most common instigators are patients, relatives of the patients and other coworkers. The most commonly experienced incidents were verbal attacks, although physical violence was also present. Younger physicians and less experience in emergency medicine were the most susceptible to both verbal and physical violence, although mitigating strategies are present, which includes reporting to senior staff member and police. However, it is also common for physicians to overlook the incidence and do nothing to address the experiences in workplace violence. Despite existing efforts to develop protect healthcare workers, there is still a need to strengthen the policies related to the protection of physicians in the emergency department in Saudi Arabia while mobilizing the health care team.

## Data Availability

The datasets generated and analyzed during this study are not publicly available due to the possibility that some information included in these data might compromise participants’ privacy but are available from the corresponding author on reasonable request.

## Abbreviations

MOH: Ministry of Health
WHO: World Health Organization
